# Interplay between Spin‐Orbit Torques and Dzyaloshinskii‐Moriya Interactions in Ferrimagnetic Amorphous Alloys

**DOI:** 10.1002/advs.202100481

**Published:** 2021-08-02

**Authors:** Yassine Quessab, Jun‐Wen Xu, Md Golam Morshed, Avik W. Ghosh, Andrew D. Kent

**Affiliations:** ^1^ Center for Quantum Phenomena, Department of Physics New York University New York NY 10003 USA; ^2^ Department of Electrical and Computer Engineering University of Virginia Charlottesville VA 22904 USA; ^3^ Department of Physics University of Virginia Charlottesville Virginia 22904 USA

**Keywords:** domain walls, Dzyaloshinskii–Moriya interactions, ferrimagnets, skyrmions, spin‐orbit torque

## Abstract

Ferrimagnetic thin films are attractive for low‐power spintronic applications because of their low magnetization, small angular momentum, and fast spin dynamics. Spin orbit torques (SOT) can be applied with proximal heavy metals that also generate interfacial Dzyaloshinskii–Moriya interactions (DMI), which can stabilize ultrasmall skyrmions and enable fast domain wall motion. Here, the properties of a ferrimagnetic CoGd alloy between two heavy metals to increase the SOT efficiency, while maintaining a significant DMI is studied. SOT switching for various capping layers and alloy compositions shows that Pt/CoGd/(W or Ta) films enable more energy‐efficient SOT magnetization switching than Pt/CoGd/Ir. Spin‐torque ferromagnetic resonance confirms that Pt/CoGd/W has the highest spin‐Hall angle of 16.5%, hence SOT efficiency, larger than Pt/CoGd/(Ta or Ir). Density functional theory calculations indicate that CoGd films capped by W or Ta have the largest DMI energy, 0.38 and 0.32 mJ m^−2^, respectively. These results show that Pt/CoGd/W is a very promising ferrimagnetic structure to achieve small skyrmions and to move them efficiently with current.

## Graphical abstract

Magnetic racetrack memories offer the possibility of increasing the density and speed of data storage. Reducing the power consumption of these devices remains a challenge. To explore materials to increase the energy efficiency associated with current‐induced motion of domain walls or skyrmions, nearly compensated ferrimagnetic CoGd alloys inserted between two heavy metals to simultaneously vary spin‐orbit torques and interfacial Dzyaloshinskii–Moriya interactions is studied.

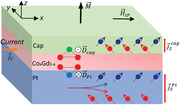

## 1 Introduction

Magnetic skyrmions are chiral spin textures with a whirling spin configuration and topological protection.^[^ ^1^ ^]^ Skyrmions behave as magnetic quasiparticles at the nanometer scale that can be created, annihilated, and displaced by applying current pulses via spin‐orbit torque (SOT).^[^ ^2^, ^3^, ^4^, ^5^, ^6^ ^]^ Skyrmions have the potential to require low energies to be moved at high velocities in the absence of an external magnetic field, which makes them attractive for spintronics applications such as racetrack memories, logic or computing devices.^[^ ^7^, ^8^, ^9^, ^10^ ^]^ In magnetic systems with broken inversion symmetry, skyrmions can be stabilized by chiral interactions such as Dzyaloshinskii–Moriya interactions (DMI).^[^ ^11^, ^12^ ^]^

Nearly compensated thin ferrimagnetic films are attractive materials due to their low dipolar fields and fast spin dynamics, and are theoretically predicted to host ultrasmall and ultrafast skyrmions at room temperature.^[^ ^13^, ^14^ ^]^ Indeed, in contrast to ferromagnetic films, the low saturation magnetization in ferrimagnets enables the formation of small skyrmions, for example sub‐20 nm skyrmions, observed in a ferrimagnetic CoGd alloy film.^[^ ^15^ ^]^ Further, ferrimagnets generally have low domain wall pinning and exhibit an angular momentum compensation point that suppresses the Walker breakdown, effectively acting as antiferromagnets and thereby allowing faster spin dynamics set by exchange interactions instead of the magnetic anisotropy.^[^ ^15^, ^16^, ^17^ ^]^ In fact, at the angular momentum compensation temperature, current‐induced domain wall velocities of up to 1000 m s^−1^ have been demonstrated in a CoGd film.^[^ ^15^ ^]^ However, a skyrmion velocity of just 50 m s^−1^ induced by SOT current pulses has been demonstrated in a ferrimagnetic multilayer.^[^ ^18^ ^]^ Thus, the fast motion of small skyrmions predicted in ferrimagnets remains elusive.

The dynamics of chiral textures such as domain walls and skyrmions is commonly described by a 1D model and associated Thiele equation^[^ ^19^ ^]^ that takes into account SOT effects and DMI.^[^ ^20^, ^21^, ^22^ ^]^ The skyrmion velocity is predicted to scale linearly with current density and depends on the SOT efficiency and the DMI strength.^[^ ^21^ ^]^ In addition, the DMI energy determines the size of the skyrmion^[^ ^13^, ^14^ ^]^ as well as its overall lifetime.

In this work, our aim is to identify a CoGd structure with the largest SOT efficiency, while maintaining an adequate DMI to ensure small metastable skyrmions. Based on the 1D model, a higher SOT efficiency lowers the current threshold to enable fast current‐induced motion of small skyrmions. Here, we investigate the SOT effects and the DMI in ferrimagnetic CoGd films as a function of the alloy composition and various heavy metals (Ta, W, and Ir) as capping layer, to simultaneously vary the spin‐Hall efficiency and the DMI strength. Our SOT switching experiments demonstrate that CoGd films capped by W and Ta exhibit the lowest switching current density, and are thus the most energy efficient for SOT‐induced manipulation of magnetization. SOT switching experiments in these films also reveal an intermediate state at low in‐plane magnetic field, which is an evidence of current‐induced motion of chiral domain walls. We attribute this metastable state to the large DMI of CoGd films capped by W and Ta. Finally, we characterize the SOT efficiency as a function of the alloy composition and capping layer by measuring the spin‐Hall angle (SHA) via spin‐torque ferromagnetic resonance (ST‐FMR), and correlate this with DMI energies obtained from density functional theory (DFT) calculations. We find that CoGd/W films have the largest SHA and DMI. Our results show that films capped by W are thus the most promising ferrimagnetic CoGd structures to achieve energy‐efficient and fast current‐induced motion of small skyrmions.

## 2 Results and Discussion

### 2.1 Samples and Magnetic Properties

The ferrimagnetic structures that we studied, Pt(6 nm)/Co_x_Gd_1‐x_(5 nm)/X (3 nm) with X = Ta, W, or Ir and *x* = 77% or 73%, were grown by DC magnetron co‐sputtering. We have previously carried out a systematic study of the DMI in Pt/CoGd/W films as a function of the thickness and stack symmetry and we demonstrated the control of the DMI in a range that allowed the formation of sub‐100 nm skyrmions.^[^ ^23^ ^]^ Typically, the ferrimagnetic stacks studied for skyrmion nucleation and motion are made with a Pt seed layer and capped by an oxide, such as Pt/CoGd/TaO_x_^[^ ^15^ ^]^ or Pt/GdFeCo/MgO.^[^ ^8^, ^18^, ^24^ ^]^ In this case, the Pt seed layer is the only source of DMI and spin currents via the spin‐Hall effect (SHE). Here, we insert the ferrimagnetic CoGd layer between two heavy metals for greater tunability of the SHA and net DMI. Ta and W are chosen for their giant SHA^[^ ^25^, ^26^, ^27^ ^]^ that is of opposite sign to Pt, leading to a maximized net SHA and thus SOT efficiency. On the other hand, the DMI due to Ir when interfaced to a ferromagnetic layer has been shown to be opposite to that of Pt, which was used to induce a larger net DMI.^[^ ^28^ ^]^

**Figure 1:**
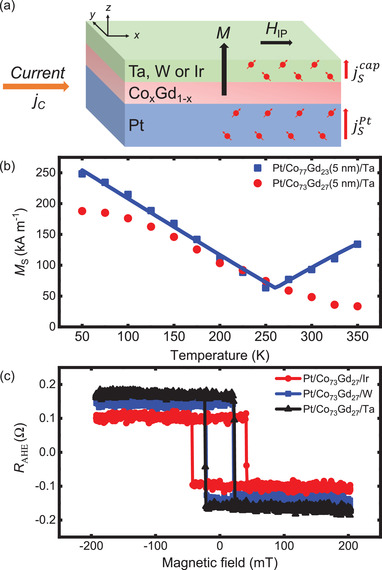
a) Schematic of the current‐induced magnetization switching via SOT where *j*_C_ is the injected charge current in the plane of the layers and parallel to the applied magnetic field *H*_IP_. Spin currents (*j*_S_) are generated at the top and bottom layer, which can exert a torque on the net CoGd magnetization (*M*). b) Temperature dependence of the saturation magnetization (*M*_S_) measured by VSM in Pt/Co_x_Gd_1 − x_/Ta for *x* = 77% and 73%. The solid blue line represents a fit to the critical power law model. c) Representative anomalous Hall resistance (*R*_AHE_) hysteresis loops measured in Pt/Co_73_Gd_27_/(Ir, W, or Ta) as a function of the out‐of‐plane magnetic field.

The magnetic properties of the CoGd films were measured by vibrating sample magnetometry (VSM) and are summarized in **Table** **1**. Here, the alloy composition was chosen in order to have a low saturation magnetization (*M*_S_) at room temperature, thus weak stray fields, which is desirable to achieve ultrasmall skyrmions,^[^ ^13^, ^14^ ^]^ as shown in Table 1. **Figure** **1**b shows the temperature dependence of *M*_S_ of Pt/Co_x_Gd_1‐x_/Ta for *x* = 77 % and 73 %. The local minimum of *M*_S_(*T*) indicates the magnetic compensation temperature (*T*_M_) at which the magnetic moments of the two antiferromagnetically coupled sublattices cancel each other out. *T*_M_ is summarized for all capping layers and alloy compositions in Table 1. The temperature dependence of the net magnetization of a ferrimagnet can be described as a function of the magnetic moments of the two sublattices by an element‐specific power law as detailed in refs. [16, 29]. The angular momentum of the Co and Gd sublattices cancel each other out at *T*_A_, the angular momentum compensation temperature. At *T*_A_, the current‐induced domain wall velocity reaches a maximum^[^ ^15^, ^29^ ^]^ as the system behaves as an antiferromagnet.^[^ ^16^ ^]^ Using the same method as Hirata *et al*.,^[^ ^29^ ^]^ by fitting *M*_S_(*T*) with a power law (solid blue line in Figure 1b), we can extract *T*_A_ for the different capping layers and CoGd alloy compositions as summarized in Table 1 (see Supporting Information). The alloy compositions were chosen in order to have *T*_A_ close to room temperature. VSM data show that the angular momentum compensation temperature of Pt/Co_77_Gd_23_/W is the closest to room temperature, making this structure an ideal candidate for fast room temperature current‐induced dynamics. Notably, *T*_M_ differs from *T*_A_ since the Co and Gd sublattices have a different g‐factor.^[^ ^15^, ^16^, ^29^ ^]^

**Table 1:** Summary of magnetic properties as a function of the capping layer and Co_x_Gd_1 − x_ alloy composition. The saturation magnetization (*M*_S_) and perpendicular magnetic anisotropy (*K*_U_) are given at room temperature, and were measured from out‐of‐plane and in‐plane magnetic hysteresis loops, respectively. The magnetic (*T*_M_) and angular momentum (*T*_A_) compensation temperatures are both determined at ± 10 K

Capping layer	*x*_Co_ [%]	*M*_S_ [kA m^−1^]	*K*_U_ [kJ m^−3^]	*T*_M_ [K]	*T*_A_ [K]
Ta	77	93	18.0	250	285
Ta	73	48	9.5	337	361
W	77	132	26.7	250	292
W	73	65	8.6	367	391
Ir	77	188	38.3	150	174
Ir	73	38	7.4	300	325

### 2.2 Spin‐Orbit Torque Switching

The skyrmion velocity is theoretically expected to linearly increase with the current density and is proportional to the SOT efficiency and DMI energy.^[^ ^21^ ^]^ The larger the current the larger the Joule heating and the energy required. Therefore, to improve the skyrmion dynamics it is desirable to achieve a higher SOT efficiency to have a lower threshold current, which also enhances the energy efficiency for skyrmion motion. As a result, our goal here is to identify a ferrimagnetic CoGd structure with the largest SOT efficiency. We investigate the SOT effects in CoGd by performing current‐induced switching experiments as a function of the alloy composition and the cap layer, which also allows us to study the influence of the DMI on the SOT switching. When a charge current (*j*_C_) flows in the adjacent heavy metal layers, due to the SHE a spin current (*j*_S_) is generated at the interface with the magnetic layer, and can exert a torque on the magnetization *M* and switch it (see Figure 1a).^[^ ^30^ ^]^ The charge‐to‐spin conversion efficiency of the SHE is characterized by the SHA (*θ*_SHE_) which is directly proportional to the SOT efficiency (*χ*_SOT_).

We performed SOT‐induced magnetization switching experiments by applying current pulses parallel to an in‐plane bias magnetic field (*H*_IP_) to break the symmetry,^[^ ^31^ ^]^ as represented in Figure 1a. The CoGd films are patterned into Hall bars to allow the detection of the magnetization direction using the anomalous Hall effect (AHE). Figure 1c shows the anomalous Hall resistance (*R*_AHE_) as a function of the out‐of‐plane magnetic field for the three different capping layers and for *x* = 77%. Similar values of *R*_AHE_ were obtained for *x* = 73%. Notably, the anomalous Hall resistance is smaller for the film capped by Ir than for Pt/CoGd(W or Ta). This can be explained by the fact that CoGd structures with Ir have a lower resistivity than W and Ta. SOT switching is realized by injecting a 110 µs current pulse, then after a 1 s delay *R*_AHE_ is measured by applying a low amplitude ms current pulse.

**Figure 2:**
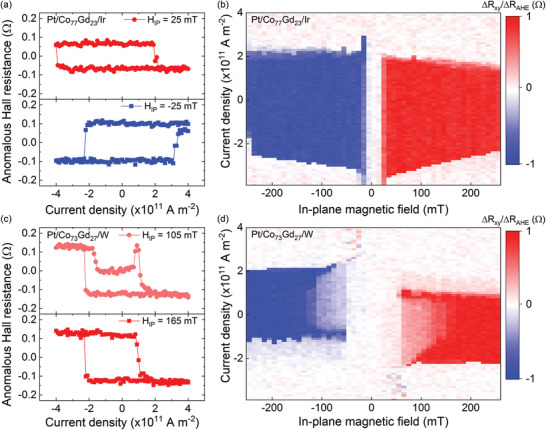
a,c) Current‐induced magnetization switching hysteresis loops for various in‐plane magnetic fields in Pt/Co_77_Gd_23_/Ir and Pt/Co_73_Gd_27_/W, respectively, and b,d) their corresponding SOT switching phase diagrams. Δ*R*_xy_ represents the relative change of the anomalous Hall resistance for a given current density and in‐plane magnetic field, and Δ*R*_AHE_ is the total anomalous Hall resistance change corresponding to full magnetization reversal as determined in Figure 1c.

Typical current‐induced magnetization switching hysteresis loops are shown in **Figure** **2**a for Pt/Co_77_Gd_23_/Ir and for *H*_IP_ = ± 25 mT. A sign reversal of the current switching hysteresis loops is observed for opposite in‐plane magnetic fields, which demonstrates that the magnetization switching is mediated by SOT.^[^ ^30^ ^]^ We then measured the SOT switching by systematically varying the in‐plane magnetic field, which allowed us to construct the SOT switching phase diagram shown in Figure 2b for Pt/Co_77_Gd_23_/Ir. The red and blue areas represent the bistable region observed in the current switching loops, as shown in Figure 2a. The boundaries of the phase diagram indicates the critical switching current density (*j*_SW_) as a function of *H*_IP_. *j*_SW_ decreases with the in‐plane magnetic field which is characteristic of SOT switching.^[^ ^32^, ^33^ ^]^ The phase diagrams were also measured for Pt/Co_77_Gd_23_/(W or Ta) (see Supporting Information). It is found that Pt/Co_77_Gd_23_/(Ir or Ta) require a bias field as low as 25 mT to induce full SOT magnetization switching. On the other hand, a field larger than 60 mT is necessary to fully switch the magnetization of Pt/Co_77_Gd_23_/W via SOT. Thermal effects in current‐induced spin‐orbit torque switching have been extensively studied in the literature and it was shown that it can lead to a reduced value of the in‐plane field necessary to initially break the system symmetry or induce a crossing of the compensation point in synthetic ferrimagnets.^[^ ^34^, ^35^, ^36^ ^]^

The emergence of an intermediate resistance state when sweeping the current was observed in the SOT switching experiments for certain CoGd structures. As seen in Figure 2c for Pt/Co_73_Gd_27_/W, an intermediate resistance plateau appeared in the current switching hysteresis loops at low in‐plane magnetic field. When increasing *H*_IP_, this intermediate state disappeared as shown in the bottom panel of Figure 2c. This metastable state can be identified in the SOT switching diagram and corresponds to the light blue and light red area in Figure 2d. In Pt/Co_x_Gd_1 − x_/W films, the metastable region was constrained to in‐plane magnetic fields below 150 and 50 mT for *x* = 73% and 77%, respectively. Films capped by Ta also exhibited a metastable state in the SOT switching, while CoGd/Ir films did not regardless of the alloy composition (see Supporting Information). Non‐uniform magnetization switching induced by SOT has already been reported in ferromagnetic films and it was found that the strong DMI was responsible for the intermediate state associated with the nucleation of chiral magnetic textures or domain wall tilting.^[^ ^37^, ^38^ ^]^ MOKE images taken at different points during the SOT switching showed current‐induced domain wall motion along or against the electron flow according to the domain wall chirality, which is responsible for the emergence of the metastable state (see Supporting Information). Since the intermediate state was not reported in CoGd/Ir films, our results suggest that the DMI is larger in CoGd films capped by W and Ta.

**Figure 3:**
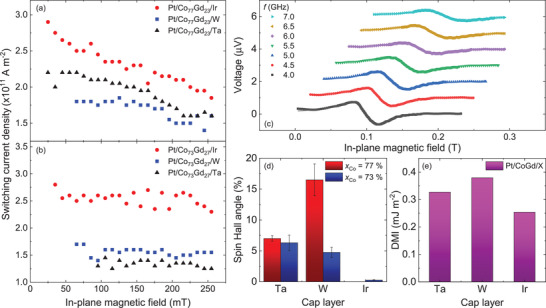
a,b) Summary of the switching current density as a function of the capping layer and in‐plane magnetic field for x=77% and 73%, respectively. c) ST‐FMR spectra of Pt/Co_73_Gd_27_/Ta. d) Summary of the spin‐Hall angle extracted from ST‐FMR measurements using the lineshape analysis method as a function of the capping layer and alloy composition. e) DMI energies determined by DFT for various capping layers in Pt/Co_75_Gd_25_.

From the SOT switching phase diagrams we measured, we can extract the switching current density for all capping layers and alloy compositions as a function of the in‐plane magnetic field. To remove any effect of asymmetries induced by misalignment of the sample with respect to the in‐plane magnetic field and in‐plane current as observed in the SOT switching phase diagrams, we define the switching current density as: $j_{\text{SW}}=\left|\frac{j_{C}^{↑↓}-j_{C}^{↓↑}}{2}\right|$, where $j_{C}^{↑↓}$ (respectively $j_{C}^{↓↑}$) is the critical current density to switch the magnetization from “up” (↑) to “down” (↓) (respectively from “down” to “up”). The critical current density is reported only when full magnetization switching is achieved. The results are summarized in **Figure** **3**a,b. *j*_SW_ ranges from 1.25 to 2.9 × 10^11^ A m^−2^, which is in the same order of magnitude as previously reported switching current densities in ferrimagnetic alloy films.^[^ ^39^, ^40^ ^]^ The switching current densities for CoGd/(W or Ta) films are always lower than CoGd/Ir films regardless of the alloy composition. The lowest switching current densities are achieved for Pt/Co_73_Gd_27_/(Ta or W). Therefore, CoGd films capped by W and Ta are the most energy‐efficient structures for SOT‐induced magnetization manipulation, and thus our results suggest that they have the largest SOT efficiency.

### 2.3 Spin‐Torque Ferromagnetic Resonance and Dzyaloshinskii‐Moriya Interactions

In order to quantify the SOT efficiency in the CoGd structures in which we studied the SOT switching, we performed spin‐torque ferromagnetic resonance (ST‐FMR) measurements. These measurements enable determination of the SHA as a function of the capping layer and alloy composition. A rf charge current at a fixed frequency (*f*) is injected into a microwire oriented at 45° with respect to the applied in‐plane magnetic field that is swept from 0 to 300 mT. The rf current generates spin currents in the bottom and top heavy metal layer interfaces that induce SOTs and Oersted field torques on the CoGd magnetization and makes it precess. The magnetization precession results in the oscillation of the device resistance due to the anisotropic magnetoresistance, which leads to a rectified dc voltage across the wire due to the spin diode effect.

Figure 3c shows the dc voltage measured as a function of the in‐plane magnetic field for several rf frequencies. We fit the ST‐FMR spectra with a sum of the derivative of Lorentzian and anti‐Lorentzian functions,^[^ ^41^, ^42^ ^]^ and extract the resonance peak amplitudes, position, and linewidth. We obtained a damping parameter *α* ≈ 0.14 that is found independent of the CoGd alloy composition and capping layer. The ratio of the Lorentzian and anti‐Lorentzian components of the ST‐FMR signal can be used to calculate the spin Hall angle $\theta_{\text{SHE}}=\left|\frac{j_{S}}{j_{C}}\right|$ using the same method as in ref. [41].

Figure 3d summarizes the extracted SHA values for different CoGd alloy compositions and capping layers. Among all of our CoGd structures, we found a maximum SHA of *θ*_SHE_ = 16.5 % ± 2.6 % for Pt/Co_77_Gd_23_/W. The SOT efficiency is defined as: $\chi_{\text{SOT}}=\theta_{\text{SHE}}\frac{\hbar }{2|e|M_{S}t_{\text{FM}}}$, where *e* is the electron charge, ℏ the reduced Planck's constant, and *t*_FM_ the thickness of the magnetic layer. Pt/Co_77_Gd_23_/W has thus the largest SOT efficiency with *χ*_SOT_ = 6.2 × 10^−8^ T A^−1^ m^2^. As seen in Figure 3d, films capped by W and Ta have a larger SHA, thus SOT efficiency, than Pt/CoGd/Ir which is consistent with the SOT switching experiments where the films capped by Ir require the largest critical current density to induce SOT switching (see Figure 3a,b). Notably, the ST‐FMR signal was too low in Pt/Co_77_Gd_23_/Ir to determine the SHA. In addition, the trends in the SHA values are consistent with the extracted switching current densities. Indeed, for *x* = 77%, we found that $\theta_{\text{SHE}}^{W}>\theta_{\text{SHE}}^{\text{Ta}}$, which is in agreement with the results of the SOT switching experiments where $j_{\text{SW}}^{W}<j_{\text{SW}}^{\text{Ta}}$ as seen in Figure 3a,b. Conversely, for *x* = 73%, $\theta_{\text{SHE}}^{W}<\theta_{\text{SHE}}^{\text{Ta}}$ which is also consistent with the fact that $j_{\text{SW}}^{W}>j_{\text{SW}}^{\text{Ta}}$.

Our ST‐FMR results in the Pt/CoGd/X series are consistent with the reported SHA values of W, Ta, and Ir in the literature.^[^ ^25^, ^26^, ^27^, ^43^ ^]^ Indeed, it was demonstrated that W and Ta have a much larger SHA than Pt and of opposite sign.^[^ ^25^, ^26^, ^27^ ^]^ Due to the chirality of the spin‐orbit interactions at the top and bottom interface in Pt/CoGd/(W or Ta), spin currents from the two adjacent layers add up leading to a maximized SOT efficiency. Conversely, it was shown that the SHA of Ir is slightly smaller than Pt and of the same sign.^[^ ^43^ ^]^ Using the same symmetry arguments, this results in a much smaller net spin current in Pt/CoGd/Ir consistent with our SOT switching and ST‐FMR results. Additionally, there are very few reports in the literature that studied SOTs in the conventional bilayer structure, Pt/ferri‐magnet, using ST‐FMR. Spin‐Hall angles ⩽ 5.5 % have been reported in Pt/GdFeCo(5 nm)/MgO films.^[^ ^18^, ^44^ ^]^

Finally, DMI are chiral interactions that arise at the interface between a magnetic layer and a non‐magnetic metal with large spin‐orbit coupling.^[^ ^11^, ^12^ ^]^ Pt/(ferro‐ or ferri‐magnet) interfaces are characterized by a large DMI strength.^[^ ^2^, ^15^, ^45^ ^]^ Similar to the SHA, by varying the capping layer in Pt/CoGd/X structures we can tune the net DMI due to the chirality of the interactions at the top and bottom CoGd interfaces. We performed first principles DFT calculations to estimate the DMI energy in the studied CoGd films using the same method that we previously developed.^[^ ^46^ ^]^ The calculated DMI energy (*D*) is plotted against the capping layer in Figure 3e. We found that W induces the highest net DMI energy (*D* ≈ 0.38 mJ m^−2^) larger than Ta and Ir. This result differs from previous reports in ferromagnetic materials where Ir induced a DMI of opposite sign of Pt leading to a maximum net DMI.^[^ ^28^, ^47^ ^]^ It appears that in a ferrimagnetic structure such as Pt/CoGd/Ir, the overall DMI contribution of Pt and Ir is of the same sign resulting in a weak net DMI as shown in Figure 3e. Additionally, we previously experimentally investigated the interfacial DMI in Pt/CoGd/W films by Brillouin light scattering and we found DMI energies of the same order of magnitude as our DFT calculations.^[^ ^23^ ^]^ Furthermore, in the SOT switching experiments, the metastable state, which was associated with chiral domain wall motion, was observed only in CoGd films capped by W and Ta and the DFT calculations confirm that these films have the largest DMI which is crucial for the formation of small skyrmions (see Figure 2b,d). Therefore, our theoretical results suggest that, indeed, the DMI may be the origin of the nonuniform magnetization configuration in the current‐induced SOT switching as discussed in ref. [37], where the authors also reported an intermediate state characterized by the nucleation of deformed skyrmions.

## 3 Conclusion

In summary, we established that Pt/Co_x_Gd_1 − x_ films capped by W and Ta are the most energy‐efficient for the current‐induced manipulation of the magnetization via SOT and that they are characterized by a large SHA of 16.5% and 6.9% for *x* = 77%, respectively. As a result, W and Ta provide the largest SOT efficiency. In addition, Pt/CoGd/(W or Ta) structures were found to have a large DMI of about 0.38 and 0.32 mJ m^−2^, respectively, which favors the emergence of a metastable state in SOT switching. This intermediate state is likely a consequence of current‐induced nucleation and motion of chiral domain walls. Besides, these ferrimagnetic films are nearly magnetically compensated at room temperature which is crucial for the formation of ultrasmall skyrmions. Additionally, Pt/Co_77_Gd_23_/W has the angular momentum compensation point (292 K) closest to room temperature which is desirable for fast current‐induced dynamics of magnetic textures at room temperature. Therefore, our results indicate that the Pt/Co_77_Gd_23_/W structure, by combining a high SOT efficiency and large DMI energy, is an ideal candidate to achieve energy‐efficient and fast current‐induced motion of small skyrmions. Our findings show a means of lowering the energy required for skyrmion dynamics induced by SOT in ferrimagnetic systems.

## 4 Experimental Section

### Film Deposition and SOT Switching

Thin films consisting of Pt (6 nm)/Co_x_Gd_1 − x_/X (3 nm) with X = Ta, W, or Ir layers were prepared by DC magnetron sputtering at room temperature and deposited onto a Si‐SiO_2_ substrate with a 4‐nm Ta adhesion layer and capped by 2‐nm of Pt to prevent oxidation. The Ar sputter pressure was set to 3 mTorr with a base pressure of about 1 × 10^−8^ Torr. The Co_x_Gd_1 − x_layer was deposited by co‐sputtering from a Co and Gd target. The alloy composition was controlled by tuning the power of the Co target and keeping the Gd target at a fixed power. The deposition rates were calibrated using a stylus profilometer. To study the SOT switching the films were patterned into a 5 × 12.5 µm^2^ Hall cross using e‐beam lithography and ion beam etching. Ti (5 nm)/Au (50 nm) contact pads were deposited by thermal evaporation and patterned by e‐beam lithography and a lift‐off process. SOT switching was realized by injecting a 110 µs long current pulse with a Keithley 6221 and the anomalous Hall resistance was measured upon a 1 s delay using a Keithley 2400. The magnetic state was reinitialiazed after a complete current hysteresis loop using an external magnetic field. The device was terminated by a GHz oscilloscope to measure the transmitted current pulse.

### Spin‐Torque Ferromagnetic Resonance

ST‐FMR was carried out by applying a rf current with a synthesized signal generator Anritsu MG2692B to a 1 × 20 µm^2^ sample at a fixed frequency. ST‐FMR devices were patterned using the same lithography process as described above. To increase the signal‐to‐noise ratio a small coil was used to modulate the magnetic field (≈ 0.2mT amplitude) at a low frequency (727Hz) co‐axial with the larger external magnetic field. The rectified signal was read out by the lock‐in amplifier. The resulting spectrum is fitted by:

(1) $$V\left(B\right)=\frac{-S\left(B-B_{0}\right)\Delta +A\left[{\left(B-B_{0}\right)}^{2}-{\left(\Delta /2\right)}^{2}\right]}{{\left[{\left(B-B_{0}\right)}^{2}+{\left(\Delta /2\right)}^{2}\right]}^{2}},$$

where *B* is the applied magnetic field, *S* and *A* are the Lorentizian and anti‐Lorentzian components, *B*_0_ is the resonance position, and Δ is the full with at half maximum. The SHA is calculated by

(2) $$\theta_{\mathrm{SHA}}=\frac{S}{A}\sqrt{1+\frac{\mu_{0}M_{\mathrm{eff}}}{B_{0}}}\frac{e\mu_{0}M_{s}t_{\mathrm{FM}}t_{\mathrm{HM}}}{\hbar },$$

where *t*_HM_ is the effective thickness of the two heavy metal layers adjacent to CoGd determined by their relative resistivities (see Supporting Information).

### DFT Calculations of Dzyaloshinskii–Moriya Interactions

The constrained magnetic moment approach in a supercell as implemented in Vienna *ab initio* simulation package within the first‐principles DFT framework was used to theoretically calculate the DMI in CoGd structures.^[^ ^48^ ^]^ The spins of CoGd layers were arranged in opposite chirality (clockwise and anticlockwise) and the total energy difference between the two configurations was taken to obtain the DMI strength as employed in previous studies.^[^ ^49^, ^50^ ^]^ Structural and computational details of these calculations are described in the recent paper by Morshed *et al*.^[^ ^46^ ^]^ For the calculations, two monolayers of Co_x_Gd_1‐x_ (0.46 nm, *x* =75%) were used and the results were scaled to a 5‐nm thick CoGd layer considering the DMI was an interfacial interaction and was inversely proportional to the thickness as experimentally demonstrated in CoGd films by Quessab et al.^[^ ^23^ ^]^

### Statistical Analysis

Saturation magnetization values were obtained from magnetic hysteresis loops (VSM data) after linear subtraction of the paramagnetic contribution of the substrate. SOT phase diagrams were measured at least three times and the current‐induced change of anomalous Hall resistance was normalized to the total change of anomalous Hall resistance induced by an out‐of‐plane magnetic field. ST‐FMR spectra were the results of ten averages and SHA values were represented with standard deviation error bars.

## Conflict of Interest

The authors declare no conflict of interest.

## Supporting information

Supporting Information

## Data Availability

The data that support the findings of this study are available from the corresponding author upon reasonable request.
