# Supplementary material for: Interplay between Spin‐Orbit Torques and Dzyaloshinskii‐Moriya Interactions in Ferrimagnetic Amorphous Alloys
Source: Adv Sci (Weinh). 2021 Aug 2;8(18):2100481. doi: 10.1002/advs.202100481 (PMC8456276; doi:10.1002/advs.202100481)
Supplement: Supplementary file 1 — Supporting Information [file ADVS-8-2100481-s001.pdf]

© 2021 Wiley-VCH GmbH

ADVANCED SCIENCE
Open Access

Supporting Information

for *Adv. Sci.*, DOI: 10.1002/advs.202100481

Interplay between Spin-Orbit Torques and Dzyaloshinskii-Moriya Interactions in Ferrimagnetic Amorphous Alloys

Yassine Quessab, Jun-Wen Xu, Md Golam Morshed, Avik W. Ghosh, and Andrew D. Kent*

Interplay between Spin-Orbit Torques and Dzyaloshinskii-Moriya Interactions in Ferrimagnetic Amorphous Alloys

Yassine Quessab,[1,*] Jun-Wen Xu,[1] Md Golam Morshed,[2] Avik W. Ghosh,[2,3] and Andrew D. Kent[1]

[1] *Center For Quantum Phenomena, Department of Physics, New York University, New York, New York 10003, USA*

[2] *Department of Electrical and Computer Engineering, University of Virginia, Charlottesville, VA 22904 USA*

[3] *Department of Physics, University of Virginia, Charlottesville, Virginia 22904, USA*

Supporting Information

CONTENTS

Temperature dependence of the saturation magnetization 2

Determination of the magnetic and angular momentum compensation temperature 2

Spin-Orbit torque switching phase diagram 3

Imaging the current-induced metastable state 4

Effective heavy metal thickness derivation for Spin-Hall angle calculation 5

References 5

TEMPERATURE DEPENDENCE OF THE SATURATION MAGNETIZATION

Figure S1 shows the temperature dependence of the saturation magnetization in Pt/Co_xGd_{1-x}/(W or Ir) for $x = 73\%$ and 77%. The values of the magnetic and angular momentum compensation points are summarized in Table I of the main manuscript.

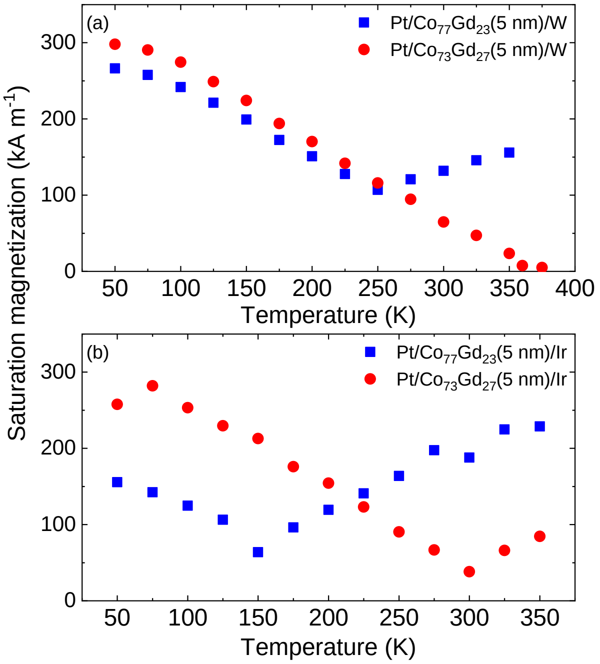

FIG. 1. Temperature dependence of the saturation magnetization measured by VSM in (a) Pt/Co_xGd_{1-x}/W and (b) Pt/Co_xGd_{1-x}/Ir with $x = 77\%$ and 73% .

DETERMINATION OF THE MAGNETIC AND ANGULAR MOMENTUM COMPENSATION TEMPERATURE

The magnetic compensation temperature (T_M) can be experimentally determined by measuring the temperature dependence of the saturation magnetization with vibrating sample magnetometry. The net CoGd saturation magnetization $M_S(T)$ can be described by an element-specific and critical power law as implemented by Kim et al.[1] based

on the magnetic moment of the two antiferromagnetically coupled sublattice:

$$M_S(T) = |M_{\mathrm{Co}}(T) - M_{\mathrm{Gd}}(T)| \tag{1}$$

where $M_{\mathrm{Co}}(T)$ and $M_{\mathrm{Gd}}(T)$ are the magnetic moments of the Co and Gd sublattice, respectively and defined as:

$$M_{\mathrm{Co}}(T) = M_{\mathrm{Co}}(0)\left(1 - \frac{T}{T_C}\right)^{\beta_{\mathrm{Co}}} \tag{2}$$

$$M_{\mathrm{Gd}}(T) = M_{\mathrm{Gd}}(0)\left(1 - \frac{T}{T_C}\right)^{\beta_{\mathrm{Gd}}} \tag{3}$$

where $M(0)$ and β are the zero temperature magnetization and the critical component of each sublattice, respectively, and T_C is the Curie temperature. We take $T_C = 450$ K, $\beta_{\mathrm{Co}} = 0.5$ and $M_{\mathrm{Co}}(0) = 1400$ kA m^{-1} according to the work of Caretta *et al.* in a similar CoGd alloy material [2]. The model parameters can be obtained by fitting Equation 1 with the experimental data as shown in Figure 1b in the main text. For Pt/$Co_{77}Gd_{23}$/Ta, we find $M_{\mathrm{Gd}}(0) = 1637$ kA m^{-1} and $\beta_{\mathrm{Gd}} = 0.68$. These values are consistent with previous results in ferrimagnetic CoGd and GdFeCo alloy films [2, 3]. The net CoGd magnetization (kA m^{-1}) can be thus written as:

$$M_S(T) = \left|1400\left(1 - \frac{T}{450}\right)^{0.5} - 1637\left(1 - \frac{T}{450}\right)^{0.68}\right| \tag{4}$$

Using the same method as Hirata *et al.*, we can deduce the magnetic and angular momentum compensation temperatures from Equation 1. At $T = T_M$, $M_S(T_M) = 0 \iff M_{\mathrm{Co}}(T_M) = M_{\mathrm{Gd}}(T_M)$. Similarly at the angular momentum compensation temperature, T_A, we have $A_{\mathrm{CoGd}}(T_A) = 0 \iff A_{\mathrm{Co}}(T_A) = A_{\mathrm{Gd}}(T_A)$, where A is the angular momentum of each sublattice, with $A_{\mathrm{Co}}(T) = \frac{M_{\mathrm{Co}}(T)}{\gamma_{\mathrm{Co}}}$ and $A_{\mathrm{Gd}}(T) = \frac{M_{\mathrm{Gd}}(T)}{\gamma_{\mathrm{Gd}}}$ where $\gamma = g\frac{\mu_B}{\hbar}$ is the gyromagnetic ratio and g the Landé factor for each sublattice. Therefore,

$$T_M = T_C\left[1 - \left(\frac{M_{\mathrm{Gd}}(0)}{M_{\mathrm{Co}}(0)}\right)^{\frac{1}{\beta_{\mathrm{Co}} - \beta_{\mathrm{Gd}}}}\right], \tag{5}$$

$$T_A = T_C\left[1 - \left(\frac{M_{\mathrm{Gd}}(0)}{M_{\mathrm{Co}}(0)}\frac{g_{\mathrm{Co}}}{g_{\mathrm{Gd}}}\right)^{\frac{1}{\beta_{\mathrm{Co}} - \beta_{\mathrm{Gd}}}}\right]. \tag{6}$$

We assume that $g_{\mathrm{Co}} = 2.05$ and $g_{\mathrm{Gd}} = 2$ [2]. Using the extracted model parameters from VSM measurements for all CoGd alloy compositions and capping layers, we can thus estimate T_A for each sample and the results are summarized in Table 1 in the main manuscript. Notably, Hirata *et al.* found a good agreement between the estimated value of T_A using this method and the experimental measurement of T_A via current-induced domain wall dynamics [3]. Finally, by combining Equation 5 and 6, the angular momentum compensation temperature can be expressed as a function of the magnetic compensation temperature:

$$T_A = T_M + T_C\left[1 - \left(\frac{g_{Co}}{g_{Gd}}\right)^{\frac{1}{\beta_{Co} - \beta_{Gd}}}\right]\left(\frac{M_{Gd}(0)}{M_{Co}(0)}\right)^{\frac{1}{\beta_{Co} - \beta_{Gd}}} \tag{7}$$

T_{M}, β_{Gd} and $M_{Gd}(0)$ are determined experimentally by VSM measurements. From Equation 7, it follows that the uncertainty in T_{A} is mainly associated with the uncertainty of T_{M}. Therefore, to the first order, both temperatures (T_{A} and T_{M}) have the same uncertainty.

SPIN-ORBIT TORQUE SWITCHING PHASE DIAGRAM

The SOT-induced magnetization switching was systematically studied as a function of the in-plane magnetic field in Pt/Co_xGd_{1-x}/(W, Ta or Ir), which allows us to construct a switching phase diagram. The phase diagrams of Pt/$Co_{77}Gd_{23}$/Ir and Pt/$Co_{73}Gd_{27}$/W are presented in the main manuscript. In Figure S2, the other diagrams for different capping layers and alloy composition are displayed.

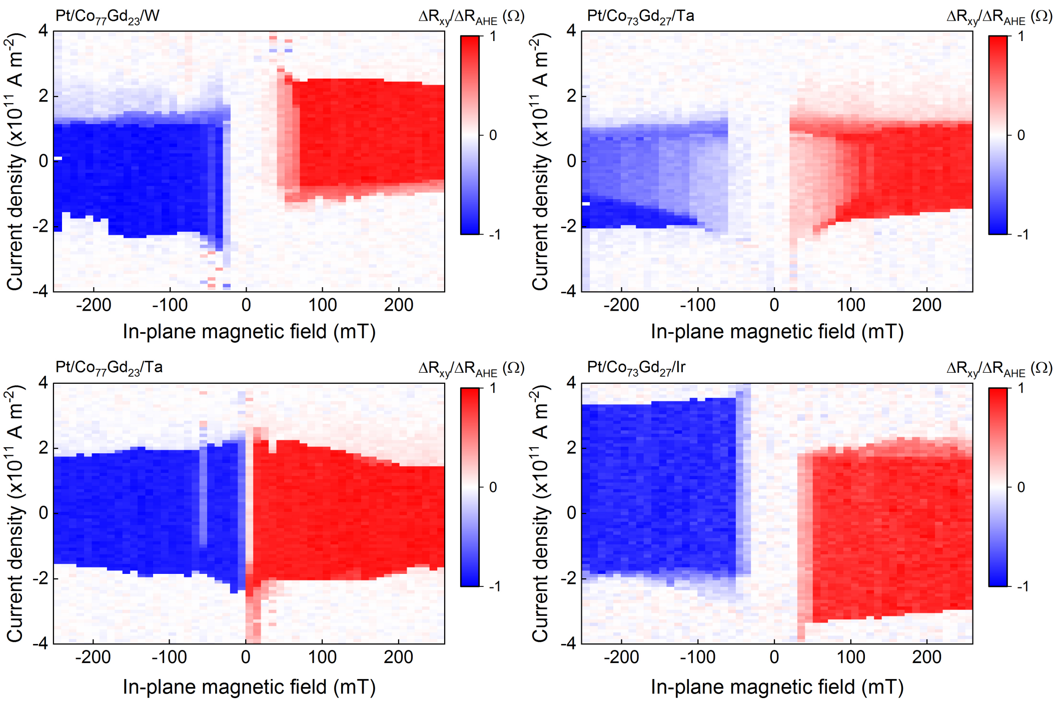

FIG. 2. Spin-orbit torque switching phase diagrams for various capping layers and alloy compositions in Pt/Co_xGd_{1-x}/(W, Ta or Ir). The red and blue areas indicate the bistable region in a SOT switching magnetization hysteresis loop. The boundary of these areas correspond to the critical switching current density as a function of the applied in-plane magnetic field. ΔR_{xy} is the relative change of the anomalous Hall resistance in the hysteresis loop for a given current density.

IMAGING THE CURRENT-INDUCED METASTABLE STATE

Using a magneto-optical Kerr effect (MOKE) microscope, we were able to observe the formation of the intermediate state that emerges in the SOT switching experiments. **Figure S3**a shows an example of an intermediate state observed in Pt/$Co_{73}Gd_{27}$/Ta with an applied in-plane magnetic field of 105 mT. The Hall cross was initially saturated in the "up" state that corresponds to $R_{AHE} = -0.2\ \Omega$ (indicated by "0"). The hysteresis loop starts with a positive current density of 4 $\times 10^{11}$ A m^{-2}. In **Figure S3**b, differential MOKE images are displayed, i.e., the images taken at different steps during the SOT switching experiment were subtracted to a reference image corresponding to a saturated state ("0") in order to enhance the magnetic contrast. The SOT current is injected along the vertical track and the anomalous Hall resistance is read using the horizontal track. The direction of the positive (resp. negative) current, j_{pos} (resp. j_{neg}), is shown in the first panel of **Figure S2**b.

The current is swept from 4 to -4×10^{11} A m^{-2} and an image is taken ("1"). In the vertical track a darker and a brighter contrast can be seen and the boundary between these two regions indicate the presence of a domain wall (DW) as shown by the white arrow in **Figure S3**b. The darker magnetic contrast represents a magnetization "down" state. Therefore, for a jump to be observed in the SOT hysteresis loop at -2×10^{11} A m^{-2} (from "up" to "down", i.e. from bright to darker contrast), a domain was nucleated in the upper part of the vertical track and the DW moved downward. The current is further reduced from -4 to $0{\times}10^{11}$ A m^{-2} and an image is taken ("2") which corresponds to the metastable. We can see in the image "2" that the DW is located in the vicinity of the center of the Hall cross. As a result, between "1" and "2" the direction of the domain wall displacement is reversed and against the current flow. Finally, the hysteresis loop is completed by increasing the current density to 4 $\times 10^{11}$ A m^{-2} and an image is taken ("3"). The Hall cross is full saturated with a brighter contrast indicative of a magnetization "down" state. Therefore, another DW emerged in the upper part of the vertical track and moved downward which resulted in the shrinkage of the darker region (magnetization "down" domain).

As a consequence, MOKE images revealed that the current-induced nucleation and then motion was responsible for the emergence of the metastable. The direction of the current-induced DW motion can be explained by considering a DW with a certain chirality provided by the DMI.

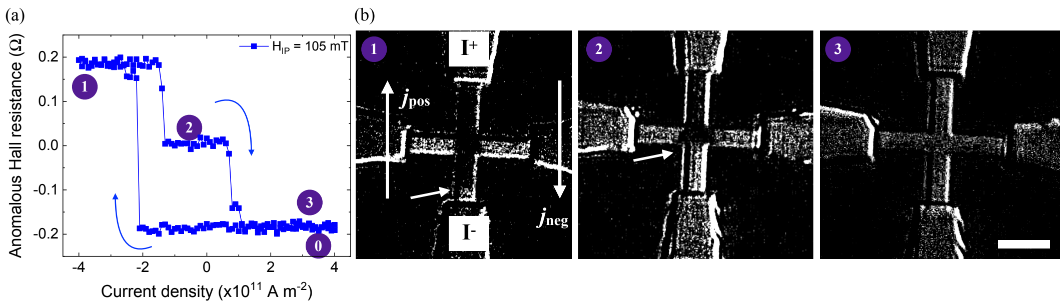

FIG. 3. (a) SOT switching hysteresis loop measured in Pt/$Co_{73}Gd_{27}$/Ta for an applied in-plane magnetic field of 105 mT. (b) Differential MOKE images taken at different step as indicated by the label number in (a). A darker (resp. brighter) contrast in the Hall cross indicate a magnetization-down (resp. -up) domain. The scale bar is 20 μm.

EFFECTIVE HEAVY METAL THICKNESS DERIVATION FOR SPIN-HALL ANGLE CALCULATION

The general formula to calculate the spin Hall angle using lineshape analysis method is given by [4],

$$\theta_{\mathrm{SHA}} = \frac{S}{A}\sqrt{1+\frac{\mu_0 M_{\mathrm{eff}}}{B_0}}\frac{e\mu_0 M_{\mathrm{s}} t_{\mathrm{FM}} t_{\mathrm{HM}}}{\hbar} \tag{8}$$

which is applicable for a ferromagnet (FM) - heavy metal (HM) bi-layer system. In the studied thin films Pt/CoGd/X, the magnetic layer is inserted between two HM layers, namely, Pt and X (X = Ta, W or Ir). Therefore, the effective HM thickness ($t_{\mathrm{HM,\ eff}}$) must be taken into account in Eq. 8. The contribution to the Oersted field from the top and bottom layer charge currents are of opposite sign. Thus, the total charge current is:

$$I_{total} = \frac{t_{\mathrm{Pt}} + t_X}{t_{\mathrm{HM,eff}}}\left(I_{\mathrm{Pt}} - I_{\mathrm{X}}\right), \tag{9}$$

where I_{Pt} and I_{X} are the current passing through Pt and each capping layer, respectively, and t the thickness of the Pt and capping layer, respectively. Using a parallel circuit model, the current flowing in each HM layer can be written as:

$$I_{\mathrm{Pt}} = I_{\mathrm{total}}\frac{\frac{t_{\mathrm{Pt}}}{\rho_{\mathrm{Pt}}}}{\frac{t_{\mathrm{Pt}}}{\rho_{\mathrm{Pt}}}+\frac{t_{\mathrm{X}}}{\rho_{\mathrm{X}}}}, \tag{10}$$

$$I_{\mathrm{X}} = I_{\mathrm{total}}\frac{\frac{t_{\mathrm{X}}}{\rho_{\mathrm{X}}}}{\frac{t_{\mathrm{Pt}}}{\rho_{\mathrm{Pt}}}+\frac{t_{\mathrm{X}}}{\rho_{\mathrm{X}}}}. \tag{11}$$

Thus, the effective HM thickness is defined as:

$$t_{\mathrm{HM,eff}} = \frac{I_{\mathrm{total}}\frac{\frac{t_{\mathrm{Pt}}}{\rho_{\mathrm{Pt}}}}{\frac{t_{\mathrm{Pt}}}{\rho_{\mathrm{Pt}}}+\frac{t_{\mathrm{X}}}{\rho_{\mathrm{X}}}} - I_{\mathrm{total}}\frac{\frac{t_{\mathrm{X}}}{\rho_{\mathrm{X}}}}{\frac{t_{\mathrm{Pt}}}{\rho_{\mathrm{Pt}}}+\frac{t_{\mathrm{X}}}{\rho_{\mathrm{X}}}}}{\frac{I_{\mathrm{total}}}{t_{\mathrm{Pt}}+t_{\mathrm{X}}}} = (t_{\mathrm{Pt}}+t_{\mathrm{X}})\frac{\frac{t_{\mathrm{Pt}}}{\rho_{\mathrm{Pt}}}-\frac{t_{\mathrm{X}}}{\rho_{\mathrm{X}}}}{\frac{t_{\mathrm{Pt}}}{\rho_{\mathrm{Pt}}}+\frac{t_{\mathrm{X}}}{\rho_{\mathrm{X}}}}, \tag{12}$$

where ρ is the resistivity of each layer, taken from the literature (Ir [5], β-W [6], β-Ta [7]).

* yassine.quessab@nyu.edu

[1] K.-J. Kim, S. K. Kim, Y. Hirata, S.-H. Oh, T. Tono, D.-H. Kim, T. Okuno, W. S. Ham, S. Kim, G. Go, Y. Tserkovnyak, A. Tsukamoto, T. Moriyama, K.-J. Lee, and T. Ono, Nature Materials **16**, 1187 (2017).
[2] L. Caretta, M. Mann, F. Büttner, K. Ueda, B. Pfau, C. M. Günther, P. Hessing, A. Churikova, C. Klose, M. Schneider, D. Engel, C. Marcus, D. Bono, K. Bagschik, S. Eisebitt, and G. S. D. Beach, Nature Nanotechnology **13**, 1154 (2018).
[3] Y. Hirata, D.-H. Kim, T. Okuno, T. Nishimura, D.-Y. Kim, Y. Futakawa, H. Yoshikawa, A. Tsukamoto, K.-J. Kim, S.-B. Choe, and T. Ono, Phys. Rev. B **97**, 220403 (2018).
[4] L. Liu, T. Moriyama, D. C. Ralph, and R. A. Buhrman, Phys. Rev. Lett. **106**, 036601 (2011).

[5] T. Fache, J. C. Rojas-Sanchez, L. Badie, S. Mangin, and S. Petit-Watelot, Physical Review B **102**, 064425 (2020).
[6] C.-F. Pai, L. Liu, Y. Li, H. W. Tseng, D. C. Ralph, and R. A. Buhrman, Applied Physics Letters **101**, 122404 (2012), https://doi.org/10.1063/1.4753947.
[7] L. Liu, C.-F. Pai, Y. Li, H. W. Tseng, D. C. Ralph, and R. A. Buhrman, Science **336**, 555 (2012), https://science.sciencemag.org/content/336/6081/555.full.pdf.
